# Are prolactin levels efficient in predicting a pituitary lesion in patients with hyperprolactinemia?

**DOI:** 10.1007/s12020-023-03678-z

**Published:** 2024-01-09

**Authors:** Emanuele Varaldo, Daniela Cuboni, Nunzia Prencipe, Luigi Simone Aversa, Michela Sibilla, Fabio Bioletto, Alessandro Maria Berton, Valentina Gasco, Ezio Ghigo, Silvia Grottoli

**Affiliations:** Division of Endocrinology, Diabetology and Metabolism; Department of Medical Sciences, University of Turin, Turin, Italy

**Keywords:** Prolactin, Hyperprolactinemia, Serial sampling, Sellar mass, Pituitary lesion, Stalk-effect

## Abstract

**Purpose:**

Data regarding the presence of a prolactin (PRL) threshold above which a pituitary magnetic resonance imaging (MRI) is mandatory in patients with hyperprolactinemia (hyperPRL) are controversial and derived primarily from studies focused on female populations. Aim of our study was to evaluate in a cohort of patients of both sexes with confirmed hyperPRL, the possible correlation between PRL values and the presence of pituitary abnormalities.

**Methods:**

We retrospectively analyzed data from patients who underwent serial PRL sampling at our Division between January 2015 and December 2022. Patients diagnosed with monomeric hyperPRL at serial sampling and with subsequent contrast-enhanced MRI results available for the pituitary region were included in the study. Exclusion criteria were prior pituitary disease, severe renal insufficiency, liver cirrhosis, uncompensated primary hypothyroidism and ongoing therapy with hyperprolactinemic drugs. Physiological causes of hyperPRL were also ruled out.

**Results:**

Out of the 1253 patients who underwent serial PRL sampling, 139 patients (101 women and 38 men) met the inclusion criteria: 106 (76.3%) patients had some form of pituitary disease, with microlesions observed in 69.8%, macrolesions in 25.5% and other findings in 4.7% of subjects. PRL values showed a modest accuracy in predicting the presence of a pituitary abnormality and the best cut-offs identified were >25 µg/L (AUC 0.767, *p* = 0.003) and >44.2 µg/L (AUC 0.697, *p* < 0.001) in men and women, respectively; however, if only patients with PRL values > 500 µg/L were excluded from the analysis, as they were already supposed to harbor a macroprolactinoma, PRL levels were not able to predict the presence of a macrolesion neither in men nor women.

**Conclusion:**

Given the high prevalence of pituitary abnormalities in patients of both sexes with hyperPRL at serial sampling, performing a pituitary imaging in all cases of hyperPRL, even if mild, appears to be a cautious choice.

## Introduction

Hyperprolactinemia (hyperPRL) is the most common pituitary disorder, with a prevalence ranging from 0.4% in an unselected normal adult population to 9-17% in women with reproductive disorders [1, 2] and it is due to an increase in circulating prolactin (PRL) values, regardless of the underlying cause. The clinical manifestations of hyperPRL mainly involve the sexual sphere, due to the impact of PRL on the gonadal axis: common symptoms are irregular menstrual cycles (oligo-amenorrhea or polymenorrhea), galactorrhea, and hirsutism in women and erectile dysfunction (ED) and gynecomastia in men, while in both sexes decrease in libido and infertility are also frequently reported [3].

Unlike other hormones secreted by the anterior pituitary gland, PRL release is regulated mainly by inhibitory mechanisms and dopamine is certainly the main actor [3, 4]. The possible causes of hyperPRL are many, generally classified into physiological, pathological (tumor and non-tumor related) and pharmacological ones [3, 5]. Moreover, macroprolactinemia (macroPRL) should always be excluded, especially in asymptomatic patients [5].

Stress certainly plays an important role among the physiological causes of hyperPRL: it has been demonstrated that the stress due to venipuncture can determine an increase in PRL values and in such cases a serial sampling can lead to normalization in up to nearly 1/3 of cases [5, 6].

On the other hand, amongst the pathological causes, the most frequent etiology of hyperPRL is a PRL-secreting pituitary adenoma, the “prolactinoma”, which represents about the 40% of functioning pituitary adenomas [7]. In such cases, PRL levels are generally markedly elevated and in fact a PRL value > 250 µg/L is classically defined as diagnostic for prolactinoma, while a value above 500 µg/L is considered indicative of macroprolactinoma [5, 8].

Once physiological and pharmacological causes have been excluded, a high PRL level in the presence of a sellar mass, however, does not always allow to diagnose with certainty a prolactinoma. Indeed, any sellar lesion (including non-functioning pituitary adenomas, parasellar masses and even the empty sella) that compresses the pituitary stalk can determine the interruption of the dopaminergic inhibitory pathways, leading to hyperPRL through the so-called “stalk effect” [3]. In these cases PRL values are often lower, almost always <100 µg/L [5, 9].

For such a reason, the degree of elevation of PRL level that necessarily excludes diagnostic investigation by focused magnetic resonance imaging (MRI) study has not been identified with certainty.

In their study, Rand et al. suggested performing an MRI only in case of PRL values > 100 µg/L [10], while on the other hand current guidelines [5, 11, 12] recommend an imaging study in all patients with persistent hyperPRL, even if mild. In this regard, previous studies [13–15] evaluating women referred for fertility problems with concomitant hyperPRL also failed to identify a PRL level threshold for which to perform a pituitary MRI, that therefore appears always recommended regardless of PRL values. However, in these studies hyperPRL was not confirmed by serial sampling, so a possible confounding factor of venipuncture stress cannot be ruled out with certainty.

All this considered, the primary outcome of our study was to evaluate, in an independent cohort of patients of both sexes referred to our medical attention and with hyperPRL at serial sampling, the possible correlation with the presence or absence of pituitary lesions, characterizing their prevalence and type in order to assess the need for pituitary imaging in all cases of persistent elevated PRL levels.

## Materials and methods

We retrospectively analyzed data of all patients who underwent serial PRL sampling at the Division of Endocrinology, Diabetology and Metabolism of the University Hospital “Città della Salute e della Scienza di Torino” (Turin, Italy) between January 2015 and December 2022. Patients were referred to our Division from physicians of all specialties and for several reasons: infertility, galactorrhea, headache and visual field anomaly, an incidental finding of hyperPRL or a pituitary lesion, hirsutism and irregular menses (females) or ED and gynecomastia (males).

All tests were conducted between 08:00 and 11:00 in a quiet room at fasting state at the endocrine investigation day unit. At baseline, a stable venous access was obtained by the insertion of an intravenous cannula into an antecubital vein, kept patent by slow infusion of 0.9% normal saline solution. Patients remained recumbent for 30 minutes when a first PRL sample was withdrawn from the indwelling cannula. After 30 more minutes a second PRL sample was collected and the lowest PRL value was considered for the analysis. In all subjects PEG precipitation for macroPRL was assayed on basal sample.

In all patients with monomeric hyperPRL (macroPRL <40%) a contrast-enhanced MRI of the sellar region was requested.

Thus, inclusion criteria were: (1) presence of monomeric hyperPRL at serial sampling and (2) availability of a subsequent imaging investigation of the pituitary region by contrast-enhanced MRI; all MRI scans were performed using a 1.5 Tesla (T) technique.

On the other hand, patients were excluded if (1) had history of previous pituitary disease; (2) severe renal insufficiency (i.e. estimated glomerular filtration rate – eGFR – calculated through the CKD-EPI [Chronic Kidney Disease Epidemiology Collaboration] equation <30 ml/min/1.73 m^2^) [16], liver cirrhosis or uncompensated primary hypothyroidism were evidenced; (3) were assuming drugs potentially associated to hyperPRL. All physiological causes of hyperPRL (i.e. pregnancy and breastfeeding) were ruled out as well.

According to the greatest diameter of the mass, pituitary lesions were divided into micro- (<10 mm) and macrolesions (≥10 mm).

The study was approved by the Local Ethics Committee and was in accordance with the principles of the Declaration of Helsinki.

### Laboratory measurements

PRL assay was determined by a sandwich immunoassay, using COBASe601 (Roche Diagnostic S.p.a. Monza, Italy) automated method. Intra- and inter-assay coefficient of variation were, respectively, 2-4.7% for low control and 1.7-4.4% for high control. Normal values in men were 3.5-15.2 µg/L and in women 5-26 µg/L; in view of the different range of normality values for the two sexes, PRL levels were also normalized for ULN (upper limit of normality).

MacroPRL was defined as positive in case of a recovery rate <40% of PRL after PEG precipitation.

### Statistical analysis

Normality was assessed using the Shapiro-Wilk test. Non-normally distributed variables and categorical data were expressed as median and interquartile range (IQR) or counts and percent, respectively. To highlight the differences between the median values of non-normally distributed variables the Mann–Whitney test was used when appropriate.

The chi-square test and the Fisher’s exact test were used to evaluate the association between binary variables, while the Spearman’s test to evaluate the correlation of continuous ones.

The receiver operating curve (ROC) analysis was used to assess PRL cut-offs and the confidence interval for the area under the curve (AUC) was calculated by a permutation analysis with 1,000 bootstrap replications.

A cut-off of P value < 0.05 was considered as statistically significant. Statistical analysis was performed using MedCalcTM^®^ (Statistical Software version 20.007, MedCalc Software Ltd, Ostend, Belgium). Figures were made using GraphPad PrismTM^®^ (version 8.0.2; GraphPad Software Inc., La Jolla, California).

## Results

Between January 1^st^ 2015 and December 31^st^ 2022, 1253 patients underwent serial PRL sampling at our Division.

One thousand one hundred and fourteen patients were subsequently excluded for several reasons: 725 patients were excluded because normoprolactinemia (normoPRL) was observed in at least one timepoint; 243 patients because they had a previous history of pituitary disease; 6 patients because macroPRL was evidenced; 108 patients were lost to follow-up and no MRI was available; 32 patients for severe renal insufficiency or for taking hyperprolactinemic drugs.

**Fig. 1:**
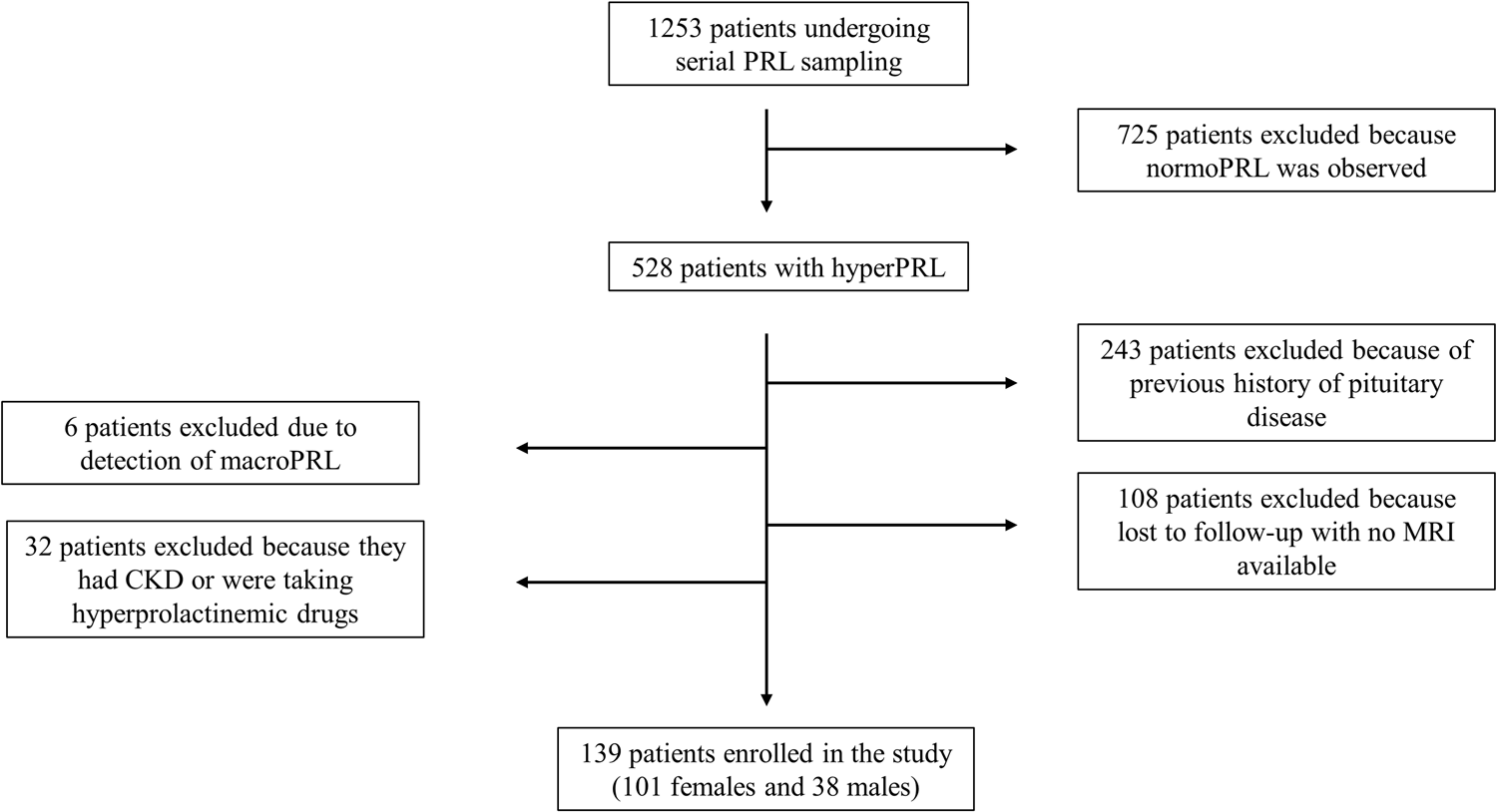
Enrollment process flow-chart. PRL Prolactin, hyperPRL Hyperprolactinemia, normoPRL Normoprolactinemia, macroPRL Macroprolactinemia, MRI Magnetic resonance imaging, CKD Chronic kidney disease

Eventually, 139 patients (38 males and 101 females) were included in the study (Fig. 1).

### Patient characteristics

The median age of the entire cohort was 37 (27-45) years; the median age of the female population was 35 (26-41) years while that of the male population was 48 (36-54) years (*p* < 0.0001). Thirteen subjects (9.4%) suffered from autoimmune primary hypothyroidism and all had a well-controlled disease. Five women (5%) had a confirmed diagnosis of polycystic ovary syndrome (PCOS) according to the Rotterdam criteria [17].

### Prolactin levels and reported symptoms

In females, the median PRL value was 54.5 µg/L (2.1xULN; IQR 36-90.9 µg/L) while in males the median value was 41.3 µg/L (2.7xULN; IQR 22.6-81.9 µg/L) without significant differences (p not significant); seven patients (4 women and 3 men) presented PRL levels >250 µg/L (range 326-12131 µg/L).

In the entire patient cohort, potentially related symptoms were found in 131 (94.9%) cases: the diagnosis of hyperPRL was incidental in 5 women and 2 men and in one woman the diagnosis was made following recurrent headache.

Alone or associated with others, the symptom most frequently reported in the female population was the alteration of the menstrual cycle (70 women, 73.7%) while hirsutism was the least reported one (3 women, 3.2%).

Thirty-six out of 38 men (94.7%) were symptomatic of hyperPRL and the most reported symptom was ED in 29 men (80.6%) while gynecomastia was reported in 10 cases (28.6%).

Counting the entire court, 15 people were referred for infertility (3 men and 12 women, 10.8%) while galactorrhea was reported in 40 subjects (39 women and 1 man, 28.8%); 22 people (20 men and 2 women, 15.8%) complained of decreased libido.

Finally, 15 subjects (7 men and 8 women, 10.8%) complained of symptoms potentially secondary to a mass effect, headache being the most reported symptom (11 subjects, 7.9%).

### Pituitary lesions

One hundred and six out of 139 patients (76.3%), and in particular 33 out of 38 males (86.8%) and 73 out of 101 females (72.3%), had some type of pituitary abnormalities (Table 1); despite not achieving statistical significance, a trend towards a greater prevalence of pituitary alterations in males compared to females was observed (*p* = 0.094). Considering the entire cohort, no difference was observed regarding age of people with and without a pituitary abnormality (*p* = 0.293) and this was confirmed even stratifying patients for sex.

**Table 1:** Radiological picture stratified on the basis of prolactin levels

**Males**						
	**PRL (µg/L)**	**Normal MRI** *n* (%)	**Microlesion** *n* (%)	**Macrolesion** *n* (%)	**Other MRI Findings**^**a**^ *n* (%)	**Total** *n*
	15.2-100	5 (17.3)	15 (51.7)	6 (20.7)	3 (10.3)	29
	100-250	0	4 (66.7)	2 (33.3)	0	6
	250-500	0	0	1 (100)	0	1
	>500	0	0	2 (100)	0	2
	Total	5	19	11	3	38
**Females**						
	26-100	28 (35.9)	43 (55.1)	6 (7.7)	1 (1.3)	78
	100-250	0	11 (57.9)	7 (36.8)	1 (5.3)	19
	250-500	0	1 (50)	1 (50)	0	2
	>500	0	0	2 (100)	0	2
	Total	28	55	16	2	101

*PRL* Prolactin, *MRI* Magnetic resonance imaging

^a^Other MRI including 3 cases of empty sella, 1 pituitary localization of sarcoidosis and 1 pituitary hyperplasia

In 74 cases (69.8%) a microlesion was evidenced while in 27 patients (25.5%) a macrolesion was appreciated; the remaining patients presented empty sella (3 subjects), a pituitary localization of sarcoidosis (1 subject) and a pituitary hyperplasia (1 subject). A macrolesion was detected in 11 males out of 30 (36.7%) and in 16 females out of 71 (22.5%) with no significant differences (*p* = 0.142). The median diameter of all sellar masses was 6 (IQR 5-10; range 3–49) mm and it was 5 (3–6) mm and 17 (11–26) mm for micro- and macrolesions, respectively.

In females, no association was found between hyperPRL-related symptoms and the presence or absence of a pituitary abnormality; in contrast, in males ED and decreased libido were associated with the presence of a pituitary alteration (*p* = 0.023 and *p* = 0.043, respectively). Finally, counting the entire cohort of patients, a significant association was observed between mass effect symptoms and the presence of a sellar mass (*p* = 0.022).

**Fig. 2:**
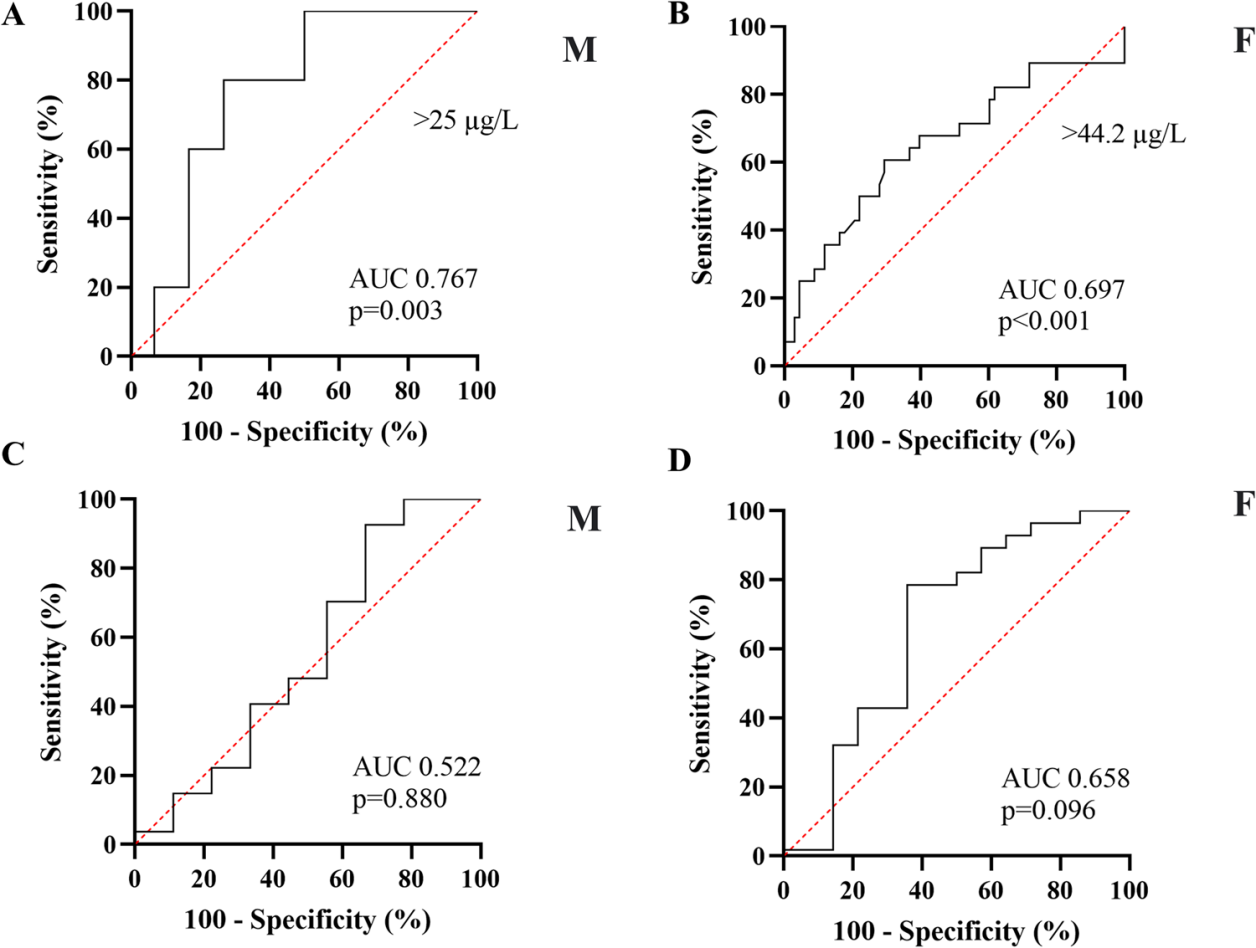
ROC (receiver operating curve) analysis showing modest accuracy for PRL in predicting the presence of a pituitary disease in men and women (2**A** and 2**B**) after excluding patients with PRL values > 250 µg/L, but poor ability in predicting the presence of a pituitary macrolesion in both sexes (2**C** and 2**D**) after excluding patients with PRL levels >500 µg/L. PRL Prolactin, AUC Area under the curve, M Males, F Females

After excluding patients with PRL values > 250 µg/L, as they were already supposed to harbor a prolactinoma [5] the ROC analysis showed a fair accuracy for PRL in predicting the presence of a pituitary disease: the best cut-offs identified were >25 (1.6xULN) µg/L (Sensitivity [Se] 73.3%, Specificity [Sp] 80%, AUC 0.767, 95% CI 0.530–0.906, *p* = 0.003) and >44.2 (1.7xULN) µg/L (Se 69.6%, Sp 64.3%, AUC 0.697, 95% CI 0.567–0.794, *p* < 0.001) in men and women, respectively (Fig. 2A, B).

Moreover, no correlation was found between PRL levels and the largest lesion diameter (*p* = 0.379) but, as expected, a significant correlation was observed if the patients with PRL values > 250 µg/L were included in the analysis as well (Spearman’s rho=0.65, *p* < 0.0001).

Finally, if only patients with PRL values > 500 µg/L were excluded from the analysis, as they were already supposed to harbor a macroprolactinoma [5, 8], the ROC analysis, neither in men nor women was able to predict the presence of a macrolesion (AUC 0.522, 95% CI 0.336-0.703, *p* = 0.880 in men; AUC 0.658, 95% CI 0.536-0.766, p = 0.096 in women) (Fig. 2C, D).

In total, 12 patients (44.4%) with a macrolesion had PRL levels <100 µg/L while 16 out of 74 (21.6%) patients with a microlesion had values > 100 µg/L and in 3 cases (4%) >200 µg/L.

Of note, amongst the women diagnosed with both PCOS and hyperPRL, 4 patients (80%) were found to have a concomitant pituitary lesion.

## Discussion

To the best of our knowledge, this is the first study to investigate in subjects of both sexes whether PRL values, once any increase due to stress is correctly excluded, can predict the presence of a pituitary disease. Specifically, our data show that after excluding PRL values that are already diagnostic for pituitary tumor-related etiology, PRL levels can predict with modest accuracy the presence of a pituitary lesion, but cannot predict a macrolesion.

Certainly, despite guidelines [5, 12] and various authors [13, 15] suggest always performing a pituitary imaging, even in the case of mild hyperPRL, this is not always applicable in real practice for several reasons, including economic and availability ones.

If on the one hand, however, the failure to diagnose a possible microlesion generally does not raise particular concern, except in rare cases characterized by hormonal hypersecretion of another nature, starting an *ex adiuvantibus* treatment with dopaminergic agonists may potentially expose patients to relevant side effects [18]. Furthermore, in certain cases this may lead to delayed detection of pituitary macrolesions which may later manifest with mass effects, resulting in the deterioration of long-term outcomes. Consequently, the identification of a PRL threshold value beyond which a MRI must necessarily be performed could allow resources to be optimized, from a cost-benefit perspective.

In 1996 it was proposed by Rand et al. [10] to use a PRL threshold >100 µg/L as a cut-off for considering imaging of the pituitary region: it is clear, however, that this value cannot be considered reliable in our series since it would have led to the loss of 74 out of 106 (69.8%) sellar masses and in particular of 12 out of 27 (44.4%) macrolesions.

Undoubtedly, we have to consider that in line with prior studies [10, 13, 14], we have identified a considerable prevalence of pituitary abnormalities in patients diagnosed with hyperPRL.

In their study, on the other hand, Souter et al. [15] observed a significantly lower prevalence of pituitary lesions with 60.9% of MRIs being negative. A distinctive aspect of this study was its exclusive focus on patients with mild-to-moderate hyperPRL (i.e. <100 µg/L) on a single sampling, although repeated on a second occasion. Consequently, it exists the possibility that a proportion of the subjects considered by such study were not “truly” hyperprolactinemic and that the reported levels could have turned normal upon serial sampling [6]. In fact, it has been shown that the highest effectiveness of serial PRL assessment occurs precisely for those patients with borderline values, while levels >94 µg/L showed 97% specificity in correctly discriminating patients with true hyperPRL [6]. In line with this, even considering only the subgroup of patients with mild-to-moderate hyperPRL in our population, the number of patients with negative MRI would still have been no more than 36% in females and not even 20% considering the male population.

As a result, patients with hyperPRL at serial sampling exhibit a notable pre-test probability of harboring a pituitary mass and a high PRL value, especially beyond 100 µg/L, as previously reported [10], is strongly indicative of an underlying pituitary cause (100% of cases in our study), once secondary causes are excluded.

On the other hand, lower PRL levels do not completely exclude the possibility of a pituitary mass, even a voluminous one, because of the aforementioned stalk effect [3]. In this context, therefore, PRL values seem more useful as a rule-in rather than a rule-out test and in light of all this, it seems to be safer to perform an MRI of the sellar region in all cases of confirmed hyperPRL, similar to what had already been suggested [13, 15]. Of note, no differences regarding the age of patients with and without a pituitary abnormality were observed, either considering the entire cohort or stratifying by sex.

Furthermore, in contrast to previous studies [13, 14], our statistical analysis excluded patients with both macroPRL, who should not undergo further diagnostic testing, as well as subjects with PRL levels indicative of a pituitary etiology ( >250 µg/L and >500 µg/L) [5, 8]. After such patients were excluded from the analysis no correlation between PRL values and greatest lesion diameter was observed, contrary to previous reports in the literature [13, 19]; however, significance was readily observed once these patients were included again in the analysis. This may be explained by the fact that our analysis was not limited to patients with a definite diagnosis of prolactinoma and that a likely large number of non-secreting lesions were present in our cohort.

Finally, our study also evaluated a significant proportion of male subjects and seems to confirm that in males idiopathic hyperPRL is not a common condition [20] and therefore always worthy of further diagnostic investigation, even for very modest elevations especially in case of symptoms such as ED.

Compared to females, men diagnosed with hyperPRL were significantly older in our cohort; moreover, lesion size seems to be typically larger [21], although a statistically significant difference was not achieved in our case.

In relation to the female cohort, on the other hand, it is noteworthy to highlight that 80% of women with a concomitant diagnosis of PCOS demonstrated an underlying pituitary lesion. Despite the limitations of our small sample size, these findings provide support for the idea that hyperPRL is not an intrinsic component of PCOS and that should not be underestimated [22].

Our study presents some strengths and limitations. One notable strength of our study is the rigorous exclusion of hyperPRL through serial sampling. Consequently, we can confidently exclude the inclusion of hyperPRL cases that were secondary to venipuncture stress among the patients analyzed. Another strength was that macroPRL was excluded in all cases. In addition, our population is larger than the aforementioned studies [13–15] and also included a large component of male subjects. Lastly, our patient cohort comprised individuals referred to our medical attention for various reasons, not solely limited to issues concerning couple infertility (just 10% of subjects). As a result, it is likely that our study population is representative of the typical clinical practice encountered by most endocrinologists.

Limitations of the study include its retrospective nature and the fact that MRIs of the sellar region were not all performed at the same center.

## Conclusion

In conclusion, guidelines recognize that PRL values > 250 µg/L and >500 µg/L indicate prolactinoma and macroprolactinoma, respectively. For lower PRL values, in subjects of both sexes with hyperPRL at serial sampling, the hormone levels seem to predict with modest accuracy the presence of a pituitary mass but not of a macrolesion.

In any case, given the high prevalence of pituitary abnormalities in this setting, the risk of missing a macrolesion does not seem to justify the refusal to perform a pituitary MRI in all cases of hyperPRL, even if mild.

## Data Availability

The data sets generated during and/or analyzed during the current study are not publicly available but are available from the corresponding author on reasonable request.
